# Diversity, Equity, and Inclusion Content on Orthopaedic Surgery Fellowship Program Websites: A Cross-Sectional, Observational Website-Content Analysis

**DOI:** 10.7759/cureus.115833

**Published:** 2026-09-05

**Authors:** Daniel Devine, Francis J Sirch, Ryan Terrany, Ashley Huezo, Suleiman Y Sudah, Allen Nicholson

**Affiliations:** 1 Orthopaedic Surgery, Rutgers Robert Wood Johnson Medical School, New Brunswick, USA; 2 Orthopedics, Monmouth Medical Center, Long Branch, USA; 3 Orthopedic Surgery, Monmouth Medical Center, Long Branch, USA; 4 Orthopaedic Surgery, Monmouth Medical Center, Long Branch, USA

**Keywords:** dei, diversity, equity, fellowship, inclusion, orthopedic training, representation

## Abstract

Introduction

The shift to virtual interviewing during the COVID-19 pandemic made program websites a central resource for residency and fellowship applicants, and applicants from groups underrepresented in medicine place particular value on programs that visibly demonstrate a commitment to diversity, equity, and inclusion (DEI). Orthopaedic surgery remains among the least diverse surgical specialties, and although professional societies such as the American Academy of Orthopaedic Surgeons (AAOS) have prioritized DEI, the extent to which these efforts are reflected on orthopaedic fellowship websites is unknown. This study evaluated the online presence of DEI-focused content across orthopaedic subspecialty fellowship program websites.

Methods

We performed a cross-sectional, observational website-content analysis. Fellowship programs across nine orthopaedic subspecialties were identified using the Fellowship and Residency Electronic Interactive Database (FREIDA) and AAOS fellowship listings and screened between March and May 2024. Programs with an accessible website were assessed for nine predefined DEI elements adapted from a previously published website-content framework. Program characteristics (geographic region, program size, and program type) were abstracted from FREIDA. Mean DEI counts were compared across subspecialties using one-way analysis of variance (ANOVA) with least-significant-difference (LSD) post-hoc testing, and adjusted models accounted for program size, type, and region.

Results

Of 450 fellowship programs identified, 448 had accessible websites and were analyzed. The mean number of DEI elements per program ranged from 4.22 (Pediatrics) to 5.76 (Foot & Ankle). The most frequently displayed elements were faculty photographs (385/448, 86%) and biographies (386/448, 86%), followed by DEI webpages (296/448, 66%) and DEI statements (293/448, 65%). Pediatrics and Shoulder & Elbow programs displayed significantly fewer DEI elements than the other subspecialties (p = 0.036). This difference persisted after adjusting for program size (p < 0.001) and program type (p < 0.001) but not for geographic region (p = 0.876).

Conclusions

Orthopaedic fellowship programs demonstrate substantial variability in the DEI content presented online, with several subspecialties underrepresenting these elements. Programs across subspecialties have an opportunity to strengthen and make more visible their DEI efforts on program websites.

## Introduction

A diverse surgical workforce is associated with improved patient outcomes, greater patient satisfaction, and healthcare teams that better reflect an increasingly diverse population [1,2]. Despite the recognized benefits of diversity and numerous initiatives led by surgical governing bodies, racial and ethnic minorities remain underrepresented among surgical subspecialty trainees [3]. Orthopaedic surgery has one of the lowest proportions of women and racial or ethnic minority trainees of any medical or surgical specialty, with these groups comprising roughly 12% of matriculating applicants, and the number of underrepresented matriculants did not meaningfully increase between 2010 and 2018 [3].

The onset of the COVID-19 pandemic in 2020 substantially altered the orthopaedic residency and fellowship application process as programs transitioned to virtual interviewing. This shift limited applicants’ ability to gauge program diversity through in-person interactions and placed greater weight on online resources for assessing the inclusiveness of a training environment. During this period, the Accreditation Council for Graduate Medical Education recommended increasing programs’ visibility, collaborating with minority interest groups, adopting more holistic candidate evaluation, and implementing tiered assessment scales [4]. Work in other specialties has shown that nearly all applicants consider a program’s online presence influential in their decision to apply [5].

The American Academy of Orthopaedic Surgeons (AAOS) has adopted diversity as a strategic goal, emphasizing targeted recruitment and the retention of women and underrepresented minorities through mentorship and leadership opportunities [6]. Even so, the recruitment of women and racial and ethnic minorities into orthopaedic residency remains low [3]. Although prior studies have examined diversity statements and initiatives on orthopaedic residency websites [7,8], a persistent shortage of mentors and peers for underrepresented trainees remains, and no study has evaluated these initiatives at the fellowship level.

In this study, we evaluated orthopaedic fellowship program websites for the presence of diversity and inclusion elements across all current orthopaedic subspecialties. We aimed to characterize the commitment of orthopaedic subspecialty education programs to diversity and inclusion as reflected online and to identify opportunities for improvement.

## Materials and methods

Study design

This cross-sectional, observational study analyzed the publicly available websites of orthopaedic fellowship programs. Because the analysis relied exclusively on public information and did not involve human participants or identifiable private data, institutional review board approval was not required. Because our outcome measure is publicly available website content, our findings characterize the diversity, equity, and inclusion (DEI)-related information that programs choose to make visible online - that is, their online representation - rather than a validated measure of a program's internal culture, climate, or actual commitment to diversity, equity, and inclusion. The analytic framework was adapted from a previously published website-content study of residency programs in obstetrics and gynecology, internal medicine, family medicine, and pediatrics [8].

Program eligibility and selection

Orthopaedic fellowship programs were identified in nine subspecialties: Shoulder & Elbow, Spine, Sports, Trauma, Pediatrics, Oncology, Hand, Foot & Ankle, and Joints (Adult Reconstruction). Programs listed in the American Medical Association's Fellowship and Residency Electronic Interactive Database (FREIDA) or the AAOS fellowship listings were eligible for inclusion. Programs were included when they had an accessible, functioning website at the time of review and were excluded when no locatable or functioning website was available. Screening occurred from March through May 2024. Among 450 identified programs, 448 had accessible websites and were included in the analysis; two Pediatrics programs were excluded because their websites were inaccessible.

Website-search procedure

Reviewers first used the website link listed in each program's FREIDA profile. When a profile did not provide a functioning link, the program website was located through a standardized Google search that combined the institution name with the terms "orthopaedic," the relevant subspecialty, and "fellowship." Each website was reviewed once during the study period. The website URL and the program's geographic region, size, and type were recorded in a shared Microsoft Excel workbook.

DEI elements and scoring definitions

Each website was evaluated for nine DEI elements selected from prior published website reviews and applicant surveys on the basis of existing validity evidence [7,8]. Reviewers coded each element dichotomously as present or absent according to predefined operational criteria. An element that could not be identified on an otherwise accessible website was coded as absent.

Fellow photographs and biographies were evaluated separately by determining whether the website displayed photographs and/or narrative biographies for any current fellows. Faculty photographs and biographies were assessed using the same present-versus-absent approach. A DEI statement was recorded when the website included an explicit stand-alone statement expressing a commitment to diversity, equity, and inclusion or an affirmative-action statement; the statement type was initially documented and then collapsed into present versus absent for analysis. A patient-population description was considered present when the program or its affiliated institution described the demographic characteristics of the population served. A DEI webpage was defined as a dedicated page that compiled DEI-specific information and resources, whereas a DEI curriculum was recorded when the website referenced or described dedicated diversity, equity, or inclusion curricular content. Support for underrepresented-in-medicine (UIM) trainees and applicants was recorded when the website described resources directed toward UIM individuals, including diversity-focused visiting-student scholarships, affinity or support groups, or community resources.

Reviewer training and agreement

Three study authors completed standardized training before data collection, using a shared codebook that included the operational definition of each DEI element, examples of present and absent findings, and guidance on common website locations for relevant information. The dichotomous present-or-absent scoring system was selected to reduce subjectivity. Two of the three trained authors (RT and AH) served as independent reviewers for all nine subspecialties: each website was scored separately by both reviewers, and discrepancies between their scores were resolved by discussion and consensus.

Program characteristics and handling of missing data

Program characteristics, including geographic region, program size (number of fellows), and program type, were abstracted from FREIDA profiles. Program type was categorized as university or university-affiliated, community, combined university/community, or military/other. Geographic regions followed the FREIDA U.S. Census division classification: New England, Middle Atlantic, East North Central, West North Central, South Atlantic, East South Central, West South Central, Mountain, and Pacific, with additional international and military categories. Programs without accessible websites were excluded rather than imputed. No imputation was performed for individual DEI elements; when an element could not be located on an accessible website, it was coded as absent.

Statistical analysis

Data were compiled in Microsoft Excel and analyzed using IBM SPSS Statistics, version 27. Descriptive statistics were used to summarize the mean number of DEI elements per subspecialty and the number of programs displaying each element. The primary unadjusted comparison of mean DEI counts across subspecialties was performed using a one-way analysis of variance with a least significant difference post hoc test. Adjusted models were then fitted to assess whether subspecialty differences persisted after accounting for program size, program type, and geographic region. Model assumptions, including approximate normality of residuals and homogeneity of variance, were evaluated using the Shapiro-Wilk and Levene tests, respectively. Because the military-affiliated category comprised a single program, which does not permit variance estimation, the affiliation comparison was repeated with this program excluded, using both a standard analysis of variance (ANOVA) and a Welch's ANOVA (which does not assume homogeneity of variance across groups), as a sensitivity analysis. For the two subspecialty comparisons in which more than one expected cell count fell below 5 (faculty photographs and faculty biographies), an exact test was additionally computed as a robustness check to the chi-square approximation. All tests were two-sided, and statistical significance was set at α = 0.05.

## Results

Of 450 fellowship programs identified, 448 had accessible websites and were analyzed (Pediatrics 40/42, Trauma 40/40, Joints 67/67, Spine 51/51, Shoulder & Elbow 34/34, Sports 92/92, Oncology 18/18, Hand 72/72, and Foot & Ankle 34/34). The presence of each DEI element by subspecialty is summarized in Table 1, and Figure 1 presents the overall percentage of programs displaying each element. The most frequently displayed elements were faculty photographs (385/448, 86%) and biographies (386/448, 86%), followed by DEI webpages (296/448, 66%) and DEI statements (293/448, 65%). The mean number of DEI elements per program ranged from 4.22 (Pediatrics) to 5.76 (Foot & Ankle).

**Table 1:** Association between program characteristics and the number of DEI elements displayed on fellowship program websites. The number of programs (N) and the mean number of diversity, equity, and inclusion (DEI) elements identified on each program website are presented by program affiliation and geographic region. Each website was assessed for nine predefined DEI elements. Continuous data are reported as mean (standard deviation [SD]). Overall differences in mean DEI-element counts were tested using one-way analysis of variance (ANOVA): affiliation, F(4, 443) = 17.87, p < 0.001; geographic region, F(9, 437) = 2.82, p = 0.010. All tests were two-sided, and p < 0.05 was considered statistically significant; an asterisk identifies statistically significant results. ANOVA, analysis of variance; DEI, diversity, equity, and inclusion; N, number of programs; SD, standard deviation.

Program Characteristic		Number of Programs	Average Number of DEI Elements (SD)	Test statistic; P value
Affiliation				F(4, 443) = 17.87; p < 0.001*
	University-based	258	5.93 (2.47)	
	Community-based	86	3.56 (2.18)	
	Military-based	1	5 (0)	
	Combined (University + Community)	89	4.66 (2.00)	
	Other	14	5.42 (3.13)	
Region				F(9, 437) = 2.82; p = 0.010*
	Mountain	31	4.80 (2.49)	
	East North Central	72	5.50 (2.37)	
	East South Central	21	5.24 (2.68)	
	International	6	1.86 (2.06)	
	Mid-Atlantic	77	5.38 (2.46)	
	New England	39	5.25 (2.38)	
	Pacific	59	5.85 (2.35)	
	South Atlantic	91	5.10 (2.52)	
	West North Central	24	5.37 (3.25)	
	West South Central	27	3.85 (2.28)	

**Figure 1:**
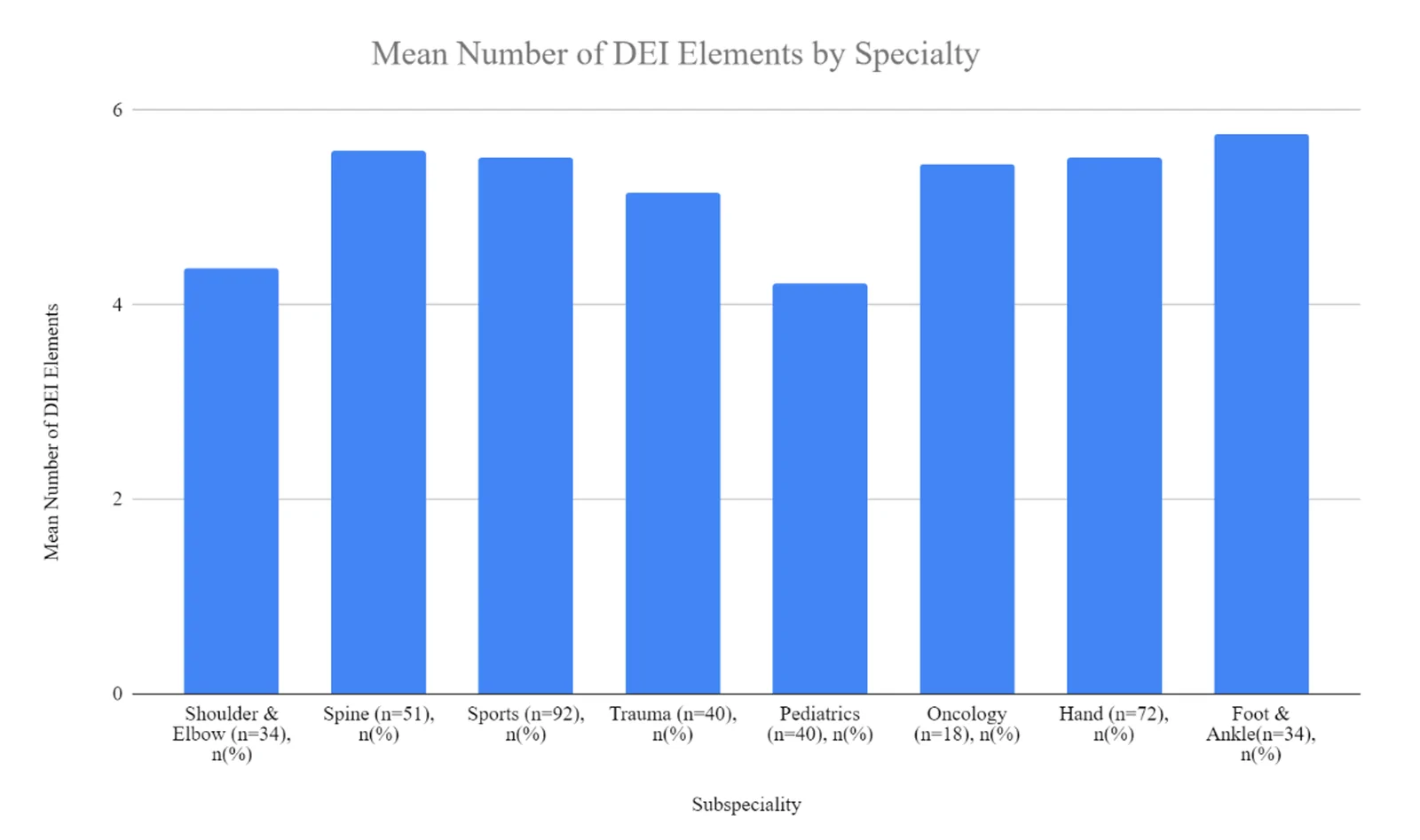
Unadjusted and adjusted mean number of DEI elements by orthopedic subspecialty. Bars represent the mean number of diversity, equity, and inclusion (DEI) elements identified on fellowship program websites within each orthopaedic subspecialty. Each website was evaluated for nine predefined DEI elements: fellow photographs, fellow biographies, faculty photographs, faculty biographies, a DEI statement, a description of the patient population, a dedicated DEI webpage, a DEI curriculum, and support for trainees and applicants underrepresented in medicine. Unadjusted values are observed means; adjusted values are model-based means after separately accounting for program size, program type, and geographic region. The number of programs (N) in each subspecialty is shown in the x-axis labels. The unadjusted overall comparison used one-way analysis of variance (ANOVA) with least significant difference post hoc testing, F(8, 439) = 2.08, p = 0.036; adjusted comparisons used regression models. All tests were two-sided, and p < 0.05 was considered statistically significant. ANOVA, analysis of variance; DEI, diversity, equity, and inclusion; N, number of programs.

Pediatrics (mean 4.22) and Shoulder & Elbow (mean 4.38) programs displayed significantly fewer DEI elements than all other subspecialties (p < 0.001); Foot & Ankle, Hand, Oncology, Trauma, Spine, and Sports programs each displayed significantly more DEI elements than Pediatrics and Shoulder & Elbow (p < 0.001; Figure 2). This difference remained significant after adjusting for program size (p < 0.001) and program type (p < 0.001) but not for geographic region (p = 0.876). The mean fellowship program size was 2.06 ± 1.34 fellows. Most programs were university-based or university-affiliated (257/448, 57.3%), and the largest share was located in the South Atlantic region (91/448, 20.3%). Notably, the military-affiliated category in Table 1 comprises a single program (N = 1); because a group of this size does not permit variance estimation, the affiliation comparison was repeated with this program excluded. Both a standard ANOVA (F(3, 443) = 23.82, p < 0.001) and a Welch’s ANOVA not assuming equal variances (F(3, 56.4) = 24.89, p < 0.001) confirmed that the affiliation finding remained robust among the four remaining groups.

**Figure 2:**
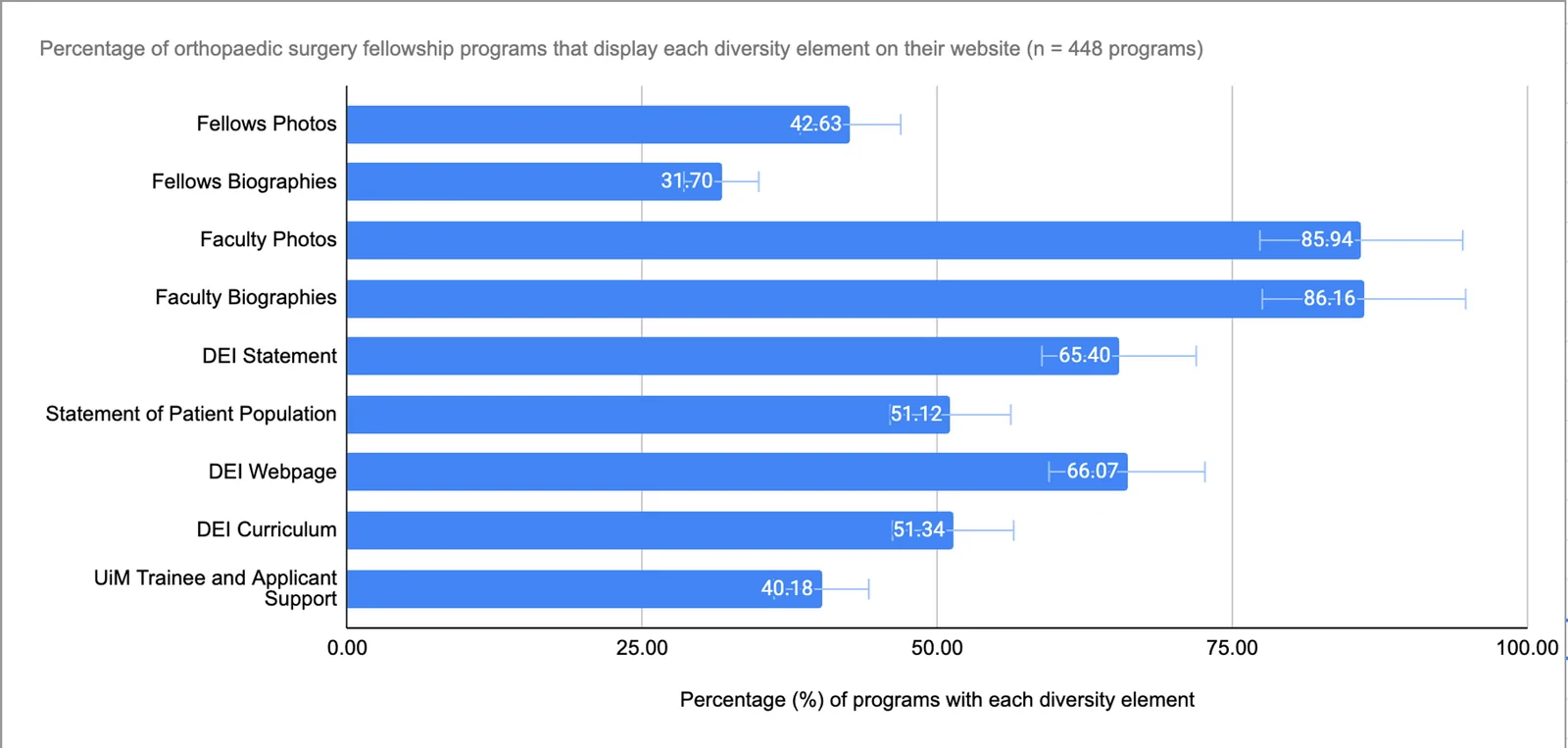
Percentage of orthopaedic surgery fellowship programs that display each diversity element on their website (n = 448 programs). Programs most often featured faculty photos and biographies. Horizontal bars represent the percentage of the 448 orthopaedic surgery fellowship program websites that displayed each of nine predefined diversity, equity, and inclusion (DEI) elements: fellow photographs, fellow biographies, faculty photographs, faculty biographies, a DEI statement, a description of the patient population served, a dedicated DEI webpage, a DEI curriculum, and support for trainees and applicants underrepresented in medicine (UIM). Data are presented as number (N) and percentage (%); values printed within the bars are percentages, and error bars are 95% confidence intervals (CIs). This figure presents descriptive statistics only; no inferential statistical test or p value is displayed. For inferential analyses in the study, two-sided p < 0.05 was considered statistically significant. CI, confidence interval; DEI, diversity, equity, and inclusion; N, number of programs; UIM, underrepresented in medicine.

Across all programs, websites contained a mean of 5.20 ± 2.53 DEI elements, and the distribution of the number of elements per program is shown in Figure 3. A stratified comparison of DEI content by program affiliation and region is presented in Table 2. Community-affiliated programs displayed significantly fewer DEI elements than university (p < 0.001), combined (p = 0.007), and other (p = 0.007) program types. By region, international (1.86 ± 2.06) and West South Central (3.85 ± 2.28) programs displayed significantly fewer DEI elements than their counterparts, whereas Pacific (5.85 ± 2.35) and East North Central (5.50 ± 2.37) programs displayed significantly more. Expected cell counts fell below 5 in two of the nine rows of Table 2 (faculty photographs, faculty biographies); exact tests for these two comparisons corroborated the chi-square results (faculty photographs: exact p = 0.038 vs. χ² p = 0.039; faculty biographies: exact p = 0.005 vs. χ² p = 0.004).

**Figure 3:**
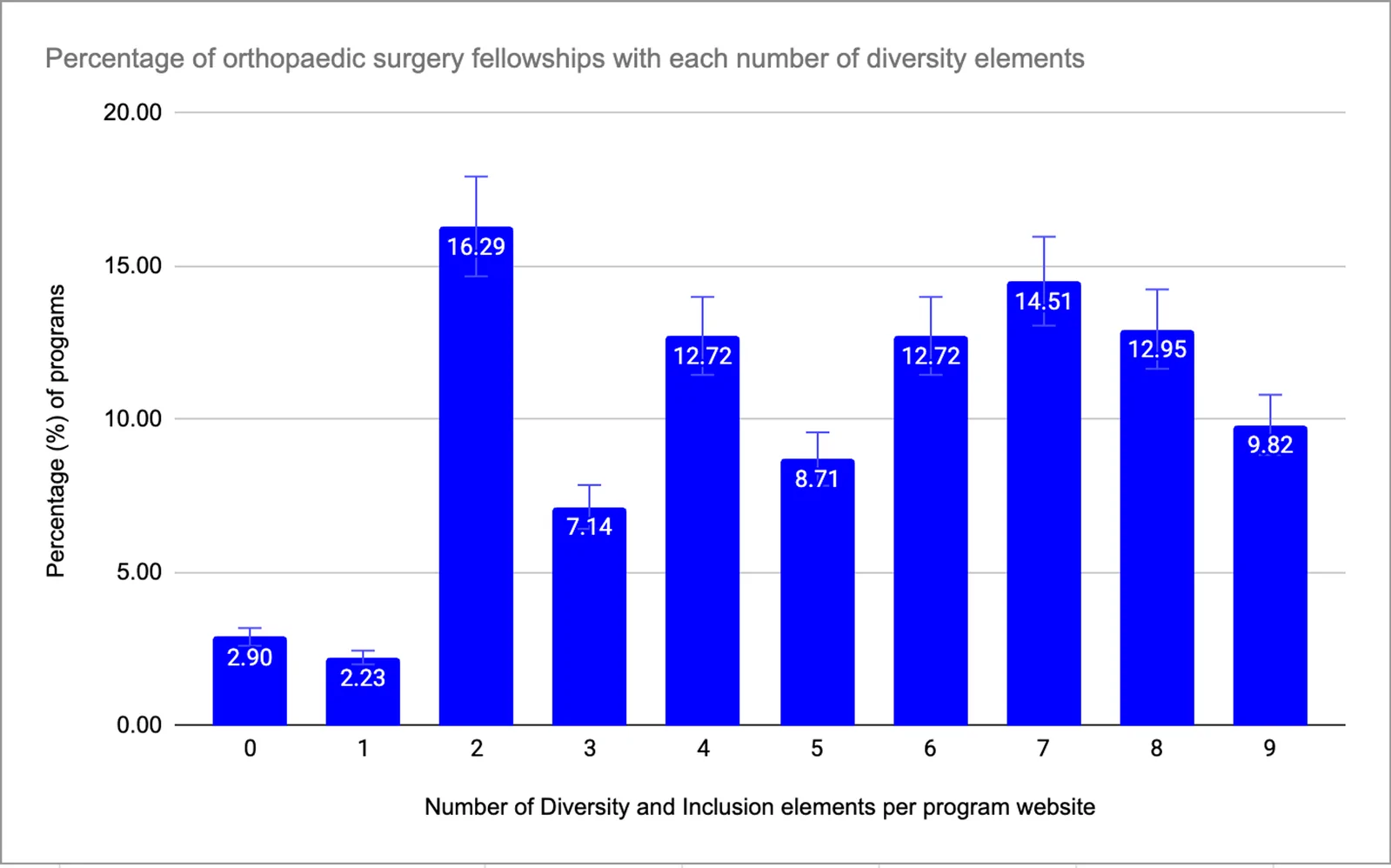
Percentage of orthopaedic surgery fellowship programs with each number of diversity elements. Of the 9 diversity elements studied, residency program websites showcased a mean of 5.20 diversity elements. Bars represent the percentage of the 448 orthopaedic surgery fellowship program websites displaying each possible number of diversity, equity, and inclusion (DEI) elements, from zero to all nine evaluated elements. Each program contributed once to the distribution according to the total number of elements identified on its website. Data are presented as number (N) and percentage (%); values printed within the bars are percentages, and error bars are 95% confidence intervals (CIs). Across all evaluated programs, the mean ± standard deviation (SD) was 5.20 ± 2.53 DEI elements. This figure presents descriptive statistics only; no inferential statistical test or p value is displayed. For inferential analyses in the study, two-sided p < 0.05 was considered statistically significant. CI, confidence interval; DEI, diversity, equity, and inclusion; N, number of programs; SD, standard deviation.

**Table 2:** Diversity, equity, and inclusion elements present by specialty: orthopedic fellowships. The table presents the number (N) and percentage (%) of fellowship program websites within each orthopaedic subspecialty that displayed each of nine predefined diversity, equity, and inclusion (DEI) elements. Specialty-specific denominators are provided in the column headings; categorical data are reported as N (%), and the final row reports the mean number of DEI elements per website. Overall categorical comparisons across the nine specialties used Pearson chi-square tests with 8 degrees of freedom. The chi-square statistics were 15.85 for fellow photographs, 10.14 for fellow biographies, 16.15 for faculty photographs, 22.36 for faculty biographies, 9.31 for DEI statements, 18.55 for patient-population descriptions, 9.77 for DEI webpages, 19.62 for DEI curricula, and 18.14 for underrepresented-in-medicine (UIM) support. The mean-count comparison used one-way analysis of variance (ANOVA), F(8, 439) = 2.08. All tests were two-sided, and p < 0.05 was considered statistically significant; an asterisk identifies statistically significant results. ANOVA, analysis of variance; Avg, average; DEI, diversity, equity, and inclusion; N, number of programs; UIM, underrepresented in medicine.

DEI Element	Joints (n=67), n(%)	Shoulder & Elbow (n=34), n(%)	Spine (n=51), n(%)	Sports (n=92), n(%)	Trauma (n=40), n(%)	Pediatrics (n=40), n(%)	Oncology (n=18), n(%)	Hand (n=72), n(%)	Foot & Ankle(n=34), n(%)	Test statistic; P value
Fellows Photos	23 (34)	8 (23)	28 (54)	47 (51)	19 (48)	12 (30)	8 (44)	31 (43)	15 (44)	χ²(8) = 15.85; p = 0.044*
Fellows Biographies	19 (28)	6 (17)	21 (41)	35 (38)	14 (35)	8 (20)	5 (28)	23 (32)	11 (32)	χ²(8) = 10.14; p = 0.257
Faculty Photos	61 (91)	24 (70)	42 (82)	82 (89)	33 (83)	35 (88)	13 (72)	62 (86)	33 (97)	χ²(8) = 16.15; p = 0.039*
Faculty Biography	60 (89)	23 (68)	46 (90)	82 (89)	32 (80)	35 (88)	12 (67)	63 (88)	33 (97)	χ²(8) = 22.36; p = 0.004*
DEI Statement	42 (62)	20 (59)	35 (68)	56 (61)	27 (68)	21 (53)	14 (77)	53 (74)	25 (74)	χ²(8) = 9.31; p = 0.276
Patient Population	29 (43)	20 (59)	30 (58)	55 (60)	19 (48)	10 (25)	10 (56)	36 (50)	20 (59)	χ²(8) = 18.55; p = 0.017*
DEI Webpages	44 (65)	20 (59)	35 (68)	56 (61)	27 (68)	22 (55)	14 (78)	50 (69)	28 (82)	χ²(8) = 9.77; p = 0.283
DEI Curricula	26 (39)	16 (47)	33 (64)	56 (61)	19 (48)	12 (30)	10 (55)	39 (54)	19 (56)	χ²(8) = 19.62; p = 0.011*
UIM Trainee and Applicant Support	21 (31)	12 (35)	15 (29)	38 (41)	16 (40)	14 (35)	12 (67)	40 (55)	12 (35)	χ²(8) = 18.14; p = 0.019*
Avg Number of DEI Elements	4.85	4.38	5.59	5.51	5.15	4.22	5.44	5.51	5.76	F(8, 439) = 2.08; p = 0.036*

## Discussion

Our study evaluated the presence of DEI elements across orthopaedic subspecialty fellowship websites available to prospective applicants. It is important to note at the outset that this outcome reflects online representation rather than a validated measure of institutional culture: programs with strong DEI practices may not prominently feature them online, and the presence of online content does not guarantee a particular lived experience for trainees. Across 448 programs, we found substantial variability in both the number and type of DEI elements displayed. On average, programs included approximately half of the evaluated DEI elements. The most common elements were faculty photographs (86%) and biographies (86%), followed by DEI webpages (66%) and DEI statements (65%). The mean number of elements varied significantly by subspecialty, from 4.22 in Pediatrics to 5.76 in Foot & Ankle; Foot & Ankle, Hand, Oncology, Trauma, Spine, and Sports programs displayed significantly more elements, whereas Pediatrics and Shoulder & Elbow programs displayed significantly fewer.

We also observed significant differences by program affiliation and region. University-based programs displayed more DEI elements than community-based programs. Program funding and administrative resources were not directly measured in this study, so we cannot determine why this difference exists; one possible, unmeasured explanation consistent with prior literature is that academic centers on average have greater administrative and research infrastructure than community sites [7-9], but this interpretation remains speculative. Regionally, Pacific and East North Central programs showed higher mean DEI counts than West South Central and international programs. This may reflect regional differences in patient and community demographics and in institutional DEI priorities, as programs serving larger or more diverse populations may place greater emphasis on visibly signaling inclusivity [8,9]. These affiliation- and region-based differences are interpretive and hypothesis-generating; the present cross-sectional design cannot establish the underlying causes, and the lower counts observed at community-based programs may reflect a relatively greater emphasis on clinical training and comparatively fewer dedicated DEI resources rather than a lesser commitment to diversity.

Multiple studies have evaluated the usability, accessibility, and content quality of residency websites and have shown that accessibility is important to how prospective applicants assess programs and make application decisions [5,10,11]. Our findings are consistent with prior website analyses in general surgery, plastic surgery, thoracic surgery, and physical medicine and rehabilitation that incorporated diversity content. A 2021 review of general, integrated thoracic, and integrated vascular surgery programs reported that only 18% of programs included DEI content on their websites [12], and a study of physical medicine and rehabilitation residency websites found that 17% met at least one of eleven DEI criteria [13]. A 2023 analysis of integrated plastic surgery programs reported an average of 3.4 of 8 predefined DEI elements [14]. Compared with these specialties, orthopaedics has been slower to integrate DEI content into its training environments: a 2022 analysis reported that only 21% of orthopaedic residency programs displayed established DEI elements online [7], compared with more than 35% in internal medicine, family medicine, and pediatrics [8]. Primary-care specialties such as family medicine have increased diversity through structured DEI strategies, suggesting that comparable approaches could benefit orthopaedic fellowships [7-9].

Although our analysis focused on fellowship programs, our findings align with the broader literature indicating opportunities to strengthen diversity and inclusion across graduate and postgraduate medical training. Whereas residency website studies have identified resident photographs and biographies as the most frequently displayed DEI element, faculty photographs and biographies were the most common elements in our fellowship cohort, while fellow photographs and biographies and trainee and applicant support were among the least common. This may reflect a shift in emphasis during the transition to fellowship toward the faculty under whom applicants will train rather than their co-trainees. Prior research indicates that strong role models, career-planning support, and mentorship are important considerations when selecting residency programs, and this mentorship may be even more influential when orthopaedic graduates select fellowship programs [9,15-17].

A recent analysis in the Journal of the American Medical Association (JAMA) Surgery reported that orthopaedic surgery had a higher proportion of White trainees and was among the least demographically diverse specialties [16]. Residents identifying as Black comprised approximately 4.1% of orthopaedic surgery residents yet accounted for a disproportionate share of attrition [16,18]. Female residents represented the smallest proportion of trainees and had the highest risk of unintended attrition relative to male peers, and trainees who identified as minorities were less likely to feel a sense of belonging and support within their programs [16]. A limited pipeline of diverse applicants and residents perpetuates a longitudinal shortage of mentors and like-minded peers for underrepresented trainees [16]. These findings informed the 2018 AAOS strategic initiative, which set diversity as a goal through targeted recruitment, transparent membership selection, retention of women and underrepresented minorities in mentorship and leadership roles, and promotion of an inclusive culture [6]. Between 2019 and 2023, this initiative was associated with steady growth in female and underrepresented participation across levels of AAOS governance and higher selection rates of these members for leadership roles [6].

This study highlights concerns regarding the visibility of diversity and inclusion content on orthopaedic subspecialty fellowship websites. The most readily achievable improvement is the continued development of comprehensive DEI webpages that transparently describe diversity efforts and opportunities for underrepresented applicants. Because website diversity content has been associated with increases in female trainees, programs should ensure their DEI efforts are clearly displayed online and emphasized in recruitment materials [9]. A recent review of diversity initiatives in surgical training found that communicating a holistic review process increased the number of underrepresented and female applicants interviewed and ranked, and that sustained mentorship programs may support the career development of underrepresented candidates [9]. Although most of this literature concerns applicants to residency, the principles can reasonably be extended to fellowship applicants.

This study has several limitations. First, the nine DEI elements are assumed to be important to applicants and reflective of a program’s commitment to diversity, equity, and inclusion, based on prior literature, resident surveys, and governing-body recommendations; these elements may not capture all aspects of diversity valued by prospective fellowship applicants. Although the individual elements were drawn from prior published website-review and applicant-survey instruments [7,8], the specific nine-element scoring framework used here has not itself been formally validated against independent measures of trainee diversity, applicant behavior, or fellowship outcomes in orthopaedic surgery, and results should be interpreted as a descriptive index of online content rather than a validated diversity-climate instrument. Second, most published applicant surveys and prior literature pertain to residency applicants rather than orthopaedic residents applying to subspecialty fellowships. Third, our analysis relied solely on publicly available website content and may not capture the full extent of a program’s DEI efforts, which applicants may also learn about through social media, networking, email, and away rotations. Finally, because scoring used a binary present/absent scale, some subjectivity in classifying less clearly defined elements is possible; to mitigate this, each website was independently scored by two reviewers (RT and AH), and discrepancies were resolved by discussion and consensus rather than quantified with a formal inter-rater reliability statistic such as Cohen’s or Fleiss’ kappa.

## Conclusions

Our study underscores an opportunity for orthopaedic fellowship programs to more clearly communicate their DEI efforts online. Although progress has been made, meaningful gaps remain in the online representation of diversity and inclusion; whether these gaps also reflect differences in underlying institutional commitment cannot be determined from a website-content analysis alone. By addressing the gaps identified here, fellowship programs can foster more inclusive environments that reflect the communities they serve, ultimately supporting underrepresented groups and enhancing the quality of medical education and patient care.

Future research should evaluate the effectiveness of specific DEI strategies in attracting and supporting a diverse cohort of trainees and faculty. Longitudinal study is also needed to examine the long-term impact of diversity initiatives on the advancement of underrepresented minorities into leadership roles in orthopaedic surgery.
